# Early Life Stress and the Fate of Kynurenine Pathway Metabolites

**DOI:** 10.3389/fnhum.2021.636144

**Published:** 2021-04-29

**Authors:** Jeremy D. Coplan, Roza George, Shariful A. Syed, Annalam V. Rozenboym, Jean E. Tang, Sasha L. Fulton, Tarique D. Perera

**Affiliations:** ^1^Department of Psychiatry, State University of New York Downstate Medical Center, Brooklyn, NY, United States; ^2^Firstox Laboratories, Irving, TX, United States; ^3^Department of Psychiatry, Yale University School of Medicine, New Haven, CT, United States; ^4^Department of Biological Sciences, Kingsborough Community College, CUNY, Brooklyn, NY, United States; ^5^Teachers College, Columbia University, New York, NY, United States; ^6^Icahn School of Medicine at Mount Sinai, New York, NY, United States; ^7^Contemporary Care, Danbury, CT, United States

**Keywords:** kynurenine (KYN), Neuroprotection, kynurenic acid (KYNA), early life stress (ELS), Anthranilic acid (AA), non human primate, Major depressive disorders, childhood adversities

## Abstract

Early life stress (ELS) precedes alterations to neuro-immune activation, which may mediate an increased risk for stress-related psychiatric disorders, potentially through alterations of central kynurenine pathway (KP) metabolites, the latter being relatively unexplored. We hypothesized that ELS in a non-human primate model would lead to a reduction of neuroprotective and increases of neurotoxic KP metabolites. Twelve adult female bonnet macaques reared under conditions of maternal variable foraging demand (VFD) were compared to 27 age- and weight-matched non-VFD-exposed female controls. Baseline behavioral observations of social affiliation were taken over a 12-week period followed by the first cerebrospinal fluid (CSF) sample. Subjects were then either exposed to a 12-week repeated separation paradigm (RSP) or assigned to a “no-RSP” condition followed by a second CSF. We used high-performance liquid chromatography for kynurenine (KYN), tryptophan, 5-hydroxyindoleacetic acid, kynurenic acid (KYNA), and anthranilic acid (ANTH) as a proxy for quinolinic acid determination. At baseline, social affiliation scores were reduced in VFD-reared versus control subjects. CSF log KYNA and log KYNA/KYN ratio were lower in VFD-reared versus control subjects. CSF log KYNA/KYN was positively correlated with CSF log ANTH in VFD only (*r* = 0.82). Controlling for log KYNA/KYN, log ANTH was elevated in VFD-reared subjects versus controls. CSF log KYNA/KYN obtained post-RSP was positively correlated with mean social affiliation scores during RSP, specifically in VFD. ELS is associated with a reduced neuroprotective and increased neurotoxic pathway products. That the two contrasting processes are paradoxically correlated following ELS suggests a cross-talk between two opposing KP enzymatic systems.

## Introduction

The role of pathogenic neuro-immune activation is increasingly recognized in serious psychiatric disorders such as major depression, bipolar disorder, and schizophrenia (Doorduin et al., 2009; Rao et al., 2010; Frick et al., 2013). During pro-inflammatory states, peripheral elevations of plasma cytokines, such as interleukin-1, interleukin-6, tumor necrosis factor-α, and interferon-γ (IFN-γ), induce central expression of the enzyme indoleamine 2,3-dioxygenase (IDO). IDO diverts central tryptophan away from serotonin biosynthesis directly into the kynurenine pathway (KP). Two opposing neuroactive KP metabolites, kynurenic acid (KYNA), which is neuroprotective, and quinolinic acid (QUIN), which is neurotoxic, are now a primary focus in our understanding of the pathogenesis of mood and anxiety disorders and certain stress-related disorders (Savitz, 2020). At “the fork in the road,” kynurenine is metabolized either to KYNA by kynurenine aminotransferases (KATs) or to 3-hydroxykynurenine (3-HK) by kynurenine-3-monooxygenase (KMO). The latter metabolic pathway leads to anthranilic acid (ANTH) and then to QUIN. Under basal conditions, most of kynurenine in the brain is metabolized to KYNA through KATs (Parrott and O’Connor, 2015). However, pro-inflammatory cytokines shift kynurenine metabolism through KMO to 3-HK and the neurotoxic pathway (Parrott et al., 2016a). Evidently, a preferential expression of either KATs or KMO may have major implications for the maintenance of affective state.

Childhood adversity may represent an independent, yet potentially preventable, risk factor for altered neuro-immune activation that persists into adulthood (Danese et al., 2007). Dysfunctional immune responsivity is cited as an important variable linking early life stress (ELS) and poor health outcomes (Frick et al., 2013). ELS is, therefore, a well-acknowledged risk factor for the development of a range of stress-related disorders that include psychological, psychosomatic, and physical conditions (Felitti and Anda, 2010). ELS has been associated with elevations of peripheral inflammatory markers in both humans (Carpenter et al., 2010) and non-human primate species (i.e., macaque) (McCormack et al., 2006). A consideration of alterations in neuroimmune signaling and KP metabolism has been primarily studied in rodents (Brenhouse et al., 2018; Allen et al., 2020).

Neuro-immune-mediated alterations of the KP catabolites have been of growing interest as a) acute KP activation may theoretically rapidly deplete tryptophan resources necessary for serotonin biosynthesis (Sublette and Postolache, 2012); b) via increases in quinolinic acid, there is an enhancement of N-methyl D-aspartate (NMDA)-mediated neurotoxicity (Brundin et al., 2016); and c) through KYNA decreases, there is a reduction of NMDA antagonist effects and subsequent compromise of regional brain neuroprotection (Kim and Won, 2017).

QUIN concentrations, the final metabolite of the neuroexcitatory arm of the KP, are elevated in the lumbar cerebrospinal fluid (CSF) of MDD patients who attempted suicide in comparison to patients with MDD without suicidality (Brundin et al., 2016). KYNA, by contrast, functions in a neuroprotective role, thereby comprising an alternate pathway for kynurenine being funneled into the neuroexcitatory pathway. KYNA demonstrates high affinity for the NMDA receptor, thereby preventing neuronal cell death in the face of glutamate excitotoxicity (Andiné et al., 1988; Leib et al., 1996). The approval of biologic agents that specifically suppress inflammation has been used quite extensively in clinical trials examining for antidepressant effects in treatment-resistant depression (TRD) and highlights a noteworthy advancement in psychopharmacology (Raison et al., 2013; Lee et al., 2020). Thus, the elucidation of the KP catabolites and brain function in psychiatric illness may enhance therapeutic opportunities, with the goal of pursuing incrementally more effective treatments that could be applied trans-diagnostically. Recent advances have elucidated a role for the immune system, which is now recognized to be an instrumental component to cause depression from neurotoxicity rather than tryptophan depletion (Wichers et al., 2005), and although modest tryptophan decreases have been associated with depression, these occur independent of IDO activity (Wichers et al., 2005).

ELS inevitably exerts epigenetic modifications that may exhibit persistent phenotypic effects across emotional development (Suderman et al., 2012). The configuration of these non-human primate epigenetic modifications may bear relevance toward the ability to maintain behavioral and physiological homeostasis in humans (McEwen, 1998, 2003; Howell and Sanchez, 2011; Hostinar and Gunnar, 2013). The novel concept of the “compensatory immune-regulatory reflex system” (CIRS) proposes that a substantial number of patients with bipolar disorder or major depression have aberrant immune-inflammatory response systems (Maes and Carvalho, 2018). Consistent with a proposed “immune gate of depression” model, early neuroimmunological development is thought to be formative to the underlying risk and development of depressive disorders (Kowalczyk et al., 2019). Non-human primate models of ELS therefore present a cogent model for providing an initial neuroimmune trigger and activation that could potentially facilitate understanding of the complex pathways to depression, anxiety disorders, and other stress-related disorders.

In this study, we seek to develop an understanding of psycho-neuro-immune development as pertains to kynurenine metabolism. The neurodevelopmental trajectory of KP metabolism and its modulation by early life experience are of clear translational significance. We hypothesized that ELS would be associated with significant alterations in key KP metabolites that suggest persistently elevated brain neurotoxicity and reduced neuroprotection. Further, we sought to demonstrate that these KP metabolite alterations would relate to behavioral profiles reflective of affective distress.

## Methods

### Subjects

Twelve adult variable foraging demand (VFD)-reared female subjects were studied. In order to provide an age- and weight-matched control, 27 non-VFD reared subjects were studied as a comparison (see Table 1). As can be seen from Table 1, there were no age or weight differences between the VFD and the non-VFD control group. All procedures were performed in careful accordance with the Guide for the Care and Use of Laboratory Animals^1^.

**TABLE 1:** Means and standard errors for ages and weights of non-human primate groups.

Rearing	*N*	Age (mean ± SE) (years)	Mass (mean ± SE) (kg)
VFD	12	14.77 ± 1.07^a^	5.80 ± 0.38^b^
Non-VFD	27	16.18 ± 0.73^a^	5.37 ± 0.26^b^

*^*a*^*F*_1, 37_ = 1.16, *p* = 0.28; ^*b*^*F*_1, 37_ = 0.84, *p* = 0.36. VFD, variable foraging demand; SE, standard error.*

### Rearing Methods

For the VFD-rearing exposure, as previously described (Coplan et al., 1996), mothers were confronted with an environment in which adequate amounts of food were available throughout but in which the amount of time and effort necessary to obtain daily rations was unpredictable (Rosenblum and Paully, 1984). The VFD maternal food procurement schedule consisted of eight 2-week blocks in which food was either readily found (low foraging demand—LFD) alternating with 2-week blocks in which food procurement entailed more time, complexity, and effort (high foraging demand—HFD). A total of four consecutive epochs were implemented—each epoch comprised a 2-week period of LFD alternated with a two-week period of HFD—which in total comprised the 16-week maternal VFD exposure experimental period. For purposes of uniformity, the introductory 2-week block was always designated as an LFD epoch. Infant age at the time of onset of the VFD procedure was approximately 3 months of age. To “vary” maternal foraging demand, a simple device, referred to as “the foraging cart,” is implemented. Mothers forage for food that can either be buried in wood chip (HFD) or left freely exposed in containers within the cart (LFD). Mothers are required to manually search for and retrieve the apportioned food by reaching through multiple apertures situated on the side of the cart. Further details of the foraging cart and the VFD procedure itself may be found in Andrews and Rosenblum (Andrews and Rosenblum, 1991).

### Observations of Adult Social Affiliation

**FIGURE 1:**
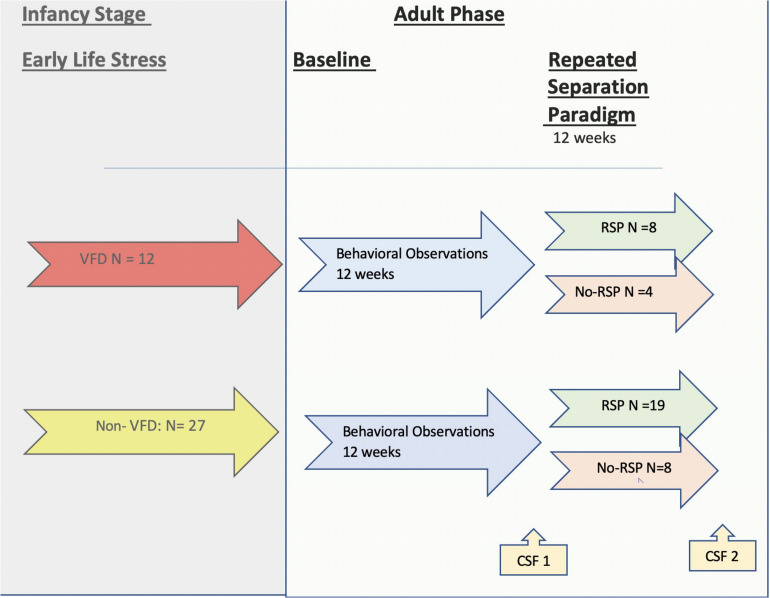
Flow chart for timeline of the current non-human primate study. A flow chart is provided depicting the two phases of the study. In the left-hand panel (in gray) is the infancy stage, during which the early life stress exposure occurs—the maternal variable foraging demand (VFD) paradigm (red arrow)—or the control condition—the non-VFD group (yellow arrow) where VFD is not imposed. In the right-hand panel side (in green) is the adult phase of the study. The baseline period comprises 12 weeks of blinded behavioral observations (blue arrows), whereas in the ensuing 12-week period, a portion of both groups are exposed to the repeated separation paradigm (RSP) (light green) or to no-RSP (light red). The number of subjects at each phase is shown in the diagram. The first cerebrospinal fluid (CSF) sample was obtained following the baseline observation period, whereas the second CSF was obtained following the RSP period (yellow arrows at bottom). Abbreviations: VFD, variable foraging demand; RSP, repeated separation paradigm; CSF, cerebrospinal fluid.

Prior to initiation of adult observation measurements, VFD subjects lived in three social groups consisting of four subjects each (*n* = 12) that had been stabilized for at least 2 months (see flow chart on Figure 1). Details of non-VFD housing arrangements are provided in the Appendix. For each experimental condition, every subject within a given social pen received the same treatment. VFD and non-VFD subjects were always housed in separate pens.

Baseline behavioral observations were conducted over a 12-week period (data on 6 weeks are available) that preceded exposure or no-exposure to a repeated separation paradigm (RSP) protocol of 12 weeks (data are available on 10 weeks), during which behavioral observations identical to the baseline period were conducted. All observation sessions were conducted at the same general time of day (from 10 am to 1 pm) to control for any potential diurnal rhythms in social behavior. The behavioral ratings were conducted by a trained technician (inter-rater reliability *λ*> 0.90), blinded to the rearing group. For 20 successive 30-s observation cycles, the presence or absence of social affiliative behaviors was identified for each animal within the social group. Thirty-second intervals were digitally timed using a tone delivered by headphones so that the tone could only be heard by the observer. Instances of hugging, grooming, receiving grooming, passive contact, and proximity were tallied and later summed to yield an overall affiliation score for each session. For the majority of subjects, a full 36 behavioral observation sessions were completed over a 12-week baseline period, generally on Mondays, Wednesdays, and Fridays (6 weeks of behavioral observations were tallied). A similar number of behavioral observations were available for the RSP (five 2-week blocks were tallied). The data is presented as the mean score/session collapsed over a 2-week block.

### Repeated Separation Paradigm

The adult RSP protocol utilized in the current study was based on a protocol developed by Higley et al. (1996) (Erickson et al., 2005). Please see the flow diagram for subjects from each rearing group that received RSP. The duration of the protocol lasted for a total of 12 weeks, during which time animals that were housed in social groups in group pens were placed in individual cages for 2.5 days (Friday evening to Monday morning) each week, leading to repeated separations and reunions. During the separation period, animals were housed in individual cages that were covered with black polyethylene covering that blocked direct visualization of other animals but not the auditory communications or olfactory cues produced by closely housed individual animals. Then, for the remainder of the week, animals were placed back into their usual social environment in the home pen. The control “no RSP” condition was non-exposed to repeated separation. All subjects housed within a given pen were either all exposed to RSP or were in the “no RSP” control condition. For VFD, four subjects in one pen underwent RSP, whereas two pens of four subjects each underwent no-RSP. Of note, there was no instance where VFD subjects were housed with non-VFD subjects. All protocols were done in compliance with the Institutional Animal Care and Use Policy of The State University of New York Downstate Health Sciences University/State University Hospital.

### CSF Methods

Subjects were taken from their home cage and placed in carrying cages, a routine procedure for cleaning purposes. For CSF sampling, subjects were released into restraint cages and intramuscular ketamine (15 mg/kg) was administered. Sedation of subjects was achieved in less than 5 min after exiting the carrying cage. Cisternal CSF sampling was performed immediately following sedation. CSF samples were then placed in Gant tubes and stored in a −70°C freezer. As part of the IACUC protocol, there is an option of administering analgesics in the event any of the animal subjects were experiencing pain or discomfort following the CSF draw. The procedure has been very well tolerated, and the subjects do not appear to experience pain or discomfort even when observed after the anesthetic and analgesic effects of ketamine have worn off.

### Kynurenine Pathway Metabolites

Liquid chromatography-tandem mass spectrometry (LC-MS/MS) was used to analyze the extraction of CSF samples, using the method described by the Clinical Laboratory and Standards Institute (Clarke et al., 2014). The samples were used to measure concentrations of CNS metabolites pertaining to tryptophan metabolism (TRP), 5-hydroxyindoleacetic acid (5-HIAA), kynurenine (KYN), kynurenic acid (KYNA), and anthranilic acid (ANTH). Linear ranges were determined to assure that the methodology used would provide accurate quantitation and reproducibility of the assay. See supplement for comprehensive description of methodology implemented. Data from our own and other laboratories indicate relative stability of monoamine metabolite concentrations (Higley et al., 1993, 1996; Kaplan et al., 1994; Shively, 1998). All laboratory personnel conducting the biochemical assays were blind to the subjects’ rearing status.

### Statistical Methods

Age and weight of animals were matched and not used as covariates. Statistica 12.0 by StatSoft^TM^ was used for analysis of all data. Outliers, which were defined as data points three deviations outside a mean that included both rearing groups, were to be excluded from the analyses. *T*-tests were performed to test for baseline characteristics. All kynurenine metabolites and their ratios were log-transformed to protect against non-homogeneity of variance. Non-parametric testing was used when appropriate. Generalized linear models were used to test for VFD rearing effects when using covariates. Factorial ANOVAs were used in the general liner model (GLM), generating an interactive term included in the overall model, specifically when comparing correlations between rearing groups. Significance was set at *p* ≤ 0.05, two-tailed.

## Results

### Age and Weight

The two groups were closely matched for age and weight and were not significantly different from each other (Table 1).

### Affiliation Scores at Baseline

Affiliation scores combined the last two of the three 2-week baseline periods and used the first baseline period as covariate. There was an overall effect for group (VFD mean ± SD: 64.67 ± 6.86, *N* = 12 vs non-VFD mean ± SD: 85.34 ± 4.75, *N* = 25; *F*_1_,_34_ = 6.01, *p* = 0.019).

### CSF Log Kynurenic Acid at Baseline

Log KYNA was significantly lower in VFD-reared subjects versus non-VFD controls at baseline (mean ± SD: 2.05 ± 0.61, *N* = 10 vs 3.32 ± 1.2, *N* = 15; *t*-value_23_ = 2.86, *p* = 0.009; Kruskal–Wallis *H*_1_,_25_ = 6.23, *p* = 0.012). There were no significant group effects for logged CSF tryptophan, kynurenine 5-HIAA, or anthranilic acid. Data related to ratios are presented below.

### CSF Log Kynurenic Acid/Kynurenine Ratio at Baseline

**FIGURE 2:**
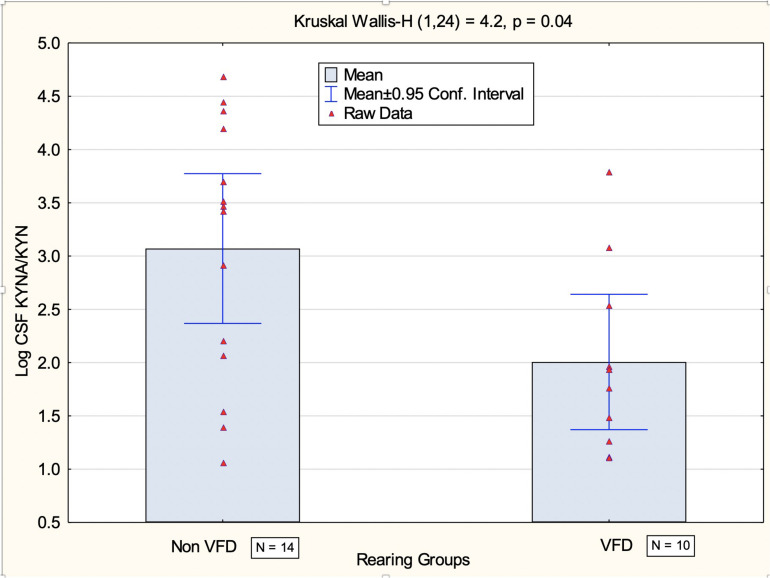
Comparison of CSF log kynurenic acid/kynurenine ratio in VFD-reared macaques and non-VFD subjects at baseline. Significant reductions were observed at baseline using the non-parametric Kruskal–Wallis test in VFD CSF log kynurenic acid/kynurenine ratio (*N* = 10) in comparison to non-VFD-exposed controls (*N* = 14). The data provide evidence for reduction of the neuroprotective arm of the kynurenine pathway following VFD rearing as kynurenic acid functions as an NMDA antagonist. Abbreviations: VFD, variable foraging demand; CSF, cerebrospinal fluid; NMDA, N-methyl D-aspartate.

CSF log KYNA/KYN ratio in VFD subjects was lower in comparison to non-VFD controls (mean ± SD: 2.00 ± 0.88, *N* = 10 versus 3.07 ± 1.21, *N* = 14; *t*-value_22_ = 2.34, *p* = 0.03) (see Figure 2 for non-parametric Kruskal–Wallis confirmation).

### Baseline CSF Kynurenine Pathway Metabolites of the Neurotoxic Arm

We utilized a GLM factorial ANOVA using CSF log ANTH as the dependent variable, CSF log KYNA/KYN as a continuous predictor variable, and rearing group as the categorical variable.

**FIGURE 3:**
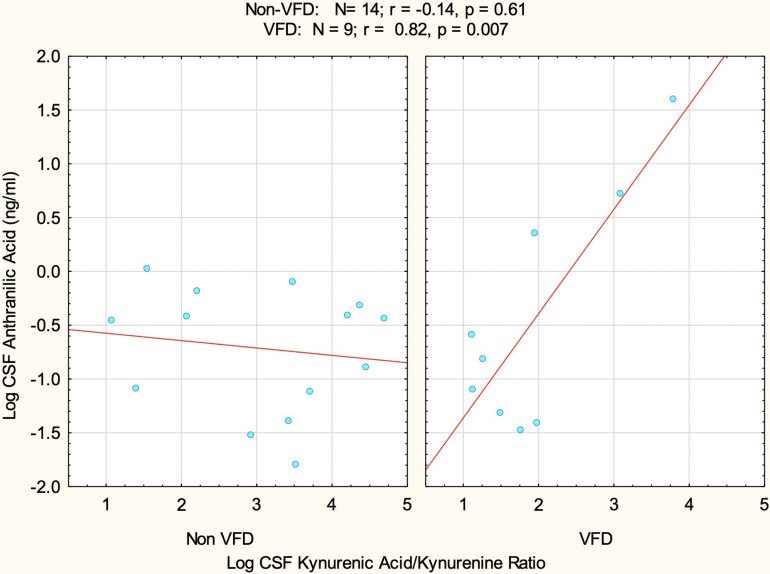
The relationship between baseline CSF log kynurenic acid/kynurenine ratio (KYNA/KYN) and CSF log anthranilic acid (ANTH) as a function of early adversity. A positive correlation was observed at baseline between the CSF log KYNA/KYN and CSF log ANTH, specifically in VFD subjects (*N* = 9) in comparison to the non-correlation in normatively reared controls (*N* = 14). There was a significant VFD*KYNA/KYN interactive effect (*F*_1_,_19_ = 13.91; *p* = 0.0014) rendering an effect size (partial *η*^2^ = 0.42) that is three-fold greater than a large effect size, deemed as 0.14. The data suggest a close synchronization of the neuroprotective and neuroxic arms—CSF ANTH serves as a proxy for the latter—within the kynurenine pathway following the VFD form of early life stress. Abbreviations: VFD, variable foraging demand; KYNA, kynurenic acid; KYN, kynurenine; CSF, cerebrospinal fluid; ANTH, anthranilic acid.

The goal was to assess the relationship between the KP neurotoxic and neuroprotective arms, taking into account that CSF log KYNA/KYN was indeed reduced in VFD at baseline. The model therefore consisted of the following variables as predictors of log ANTH: VFD grouping, log KYN/KYNA_ratio_, and the interactive term VFD^∗^log KYN/KYNA ratio. There was an overall VFD effect (*F*_1_,_19_ = 6.97; *p* = 0.016), indicating that when controlling for log KYNA/KYN ratio, VFD exhibited elevations of log ANTH [mean (95% confidence interval, CI): −0.44, (−0.88 to −0.01); *N* = 9] in comparison to non-VFD [mean (95% CI): −0.72 (−1.07 to −0.37); *N* = 14]. There was a significant effect for log KYNA/KYN ratio prediction of log ANTH (*F*_1_,_19_ = 10.47; *p* = 0.004) in a positive direction, when rearing groups are combined. There was, however, a marked VFD^∗^log KYNA/KYN interactive effect (*F*_1_,_19_ = 13.91; *p* = 0.0014) reflective of a positive correlation between log KYNA/KYN and log ANTH in VFD (*r* = 0.82, *p* = 0.007; *N* = 9) in comparison to non-VFD-reared controls (*r* = −0.14; *p* = 0.61, *N* = 14) (Figure 3). To put the latter interactive effect into context, an effect size was assessed by partial *η*^2^ of 0.42 (Cohen, 1973), where a large effect size is deemed 0.14, which places the current effect size at three-fold greater than a large effect size.

### CSF Log Kynurenic Acid/Kynurenine Ratio in Response to Repeated Separation Paradigm

**FIGURE 4:**
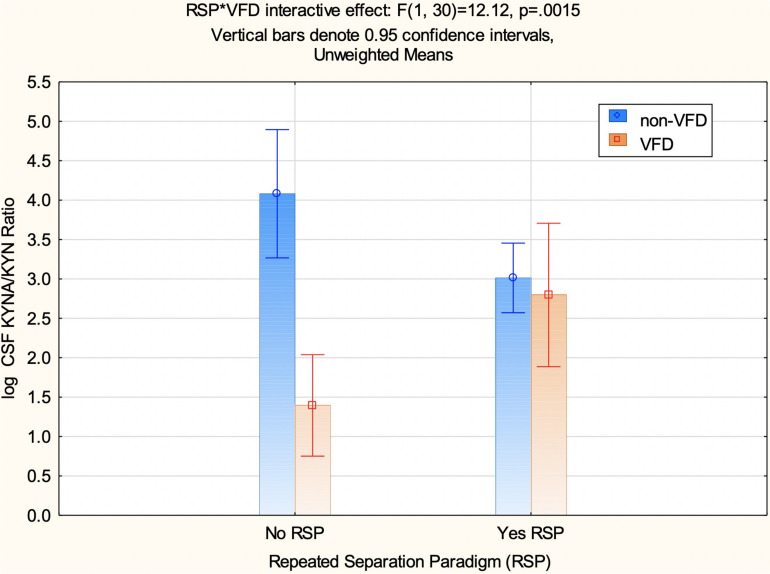
CSF log kunurenic acid/kynurenine ratio in response to the repeated separation paradigm as a function of variable foraging demand exposure during early life. There is a repeated separation paradigm (RSP)*VFD interactive effect for CSF log KYNA/KYN obtained from the CSF following the RSP phase (*F*_1_,_30_ = 12.12; *p* = 0.0016) (see Figure 1). CSF log KYNA/KYN is lower in non-VFD subjects who were exposed to the RSP (*N* = 17) in comparison to those who did not (*N* = 5; *p* = 0.0001), whereas VFD exposed to the RSP (*N* = 8) exhibited significantly increased CSF log KYNA/KYN ratios in comparison to no-RSP-exposed VFD subjects (*N* = 4; *p* = 0.04). Non-VFD unexposed to RSP exhibited exhibited greater CSF log KYNA/KYN in comparson to VFD unexposed to RSP (*p* = 0.00001). By contrast, post-exposure to RSP, VFD and non-VFD exposed are not distinguishable (*p* = 0.66). Abbreviations: VFD, variable foraging demand; KYNA, kynurenic acid; KYN, kynurenine; CSF, cerebrospinal fluid; RSP, repeated separation paradigm.

A factorial ANOVA was performed including the following variables: CSF log KYN/KYN as the independent measure and VFD grouping, RSP exposure, and a VFD^∗^RSP interactive effect (Appendix Table 1). It was not possible to integrate baseline CSF log KYNA/KYN ratio data into a repeated measure design using GLM, as values for non-VFD no-RSP exposure were not available (see Appendix for missing KP metabolite values). An overall VFD effect was observed (*F*_1_,_30_ = 16.73, *p* = 0.0003) consistent with the view that CSF log KYNA/KYN ratio is reduced in VFD irrespective of whether subjects were exposed to the RSP or not. Although no overall effect of the RSP on CSF log KYNA/KYN was evident (*F*_1_,_30_ = 0.22, *p* = 0.64), a significant effect of VFD^∗^RSP interactive effect for CSF log KYNA/KYN was observed (*F*_1_,_30_ = 12.12, *p* = 0.0016) (see Figure 4). Non-VFD subjects exhibit decreases in CSF log KYNA/KYN ratio post-RSP in comparison to no RSP exposure [mean (95% CI): no RSP: 4.08 (3.26 to 4.89), *N* = 5 versus RSP: 3.01 (2.57 to 3.45), *N* = 17; *post hoc* least square difference (LSD); *p* < 0.025]. By comparison, VFD subjects exposed to RSP exhibit increases in CSF log KYNA/KYN in comparison to VFD with no RSP exposure [mean (95% CI): no RSP: 1.39 (0.75 to 2.03); *N* = 8 versus RSP: 2.79 (1.88 to 3.70); *N* = 4; *post hoc* LSD; *p* = 0.04]. CSF log KYNA/KYN ratio of VFD subjects exposed to RSP was not distinguishable from non-VFD exposed to RSP (see Figure 4). A scatterplot with error bars using 95% CIs of CSF log KYNA/KYN ratios is presented in the Appendix to examine a comparison of values obtained from the first to the second CSF tap (see Appendix Figure 1 and flow chart in Figure 1).

### Relationship of CSF Log Kynurenic Acid/Kynurenine Ratio to Social Affiliative Behavior After Repeated Separation Paradigm as a Function of Variable Foraging Demand Rearing

**FIGURE 5:**
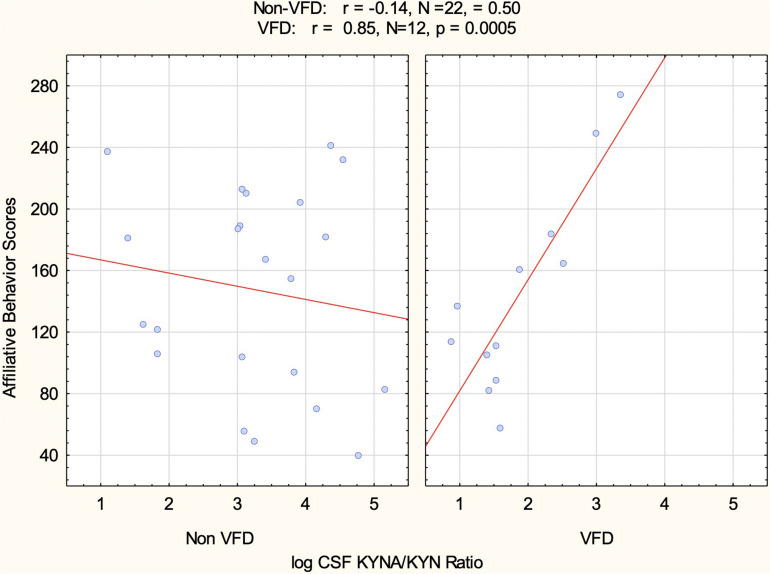
CSF log kynurenic acid/kynurenine ratio correlation with social affiliation observed during repeated separation paradigm as a function of the variable foraging demand form of early life stress. Only in VFD was there a positive relationship between social affiliation cores and the neuroprotective index represented by CSF KYNA/KYN. The positive relationship was in the expected direction. For any increment in VFD CSF log KYNA/KYN, there was a commensurate increase in social affiliation. Of note, this was the affiliation behavioral scores of the second of the five 2-week behavioral blocks (see *RESULTS* for further information). Abbreviations: VFD, variable foraging demand; KYNA, kynurenic acid; KYN, kynurenine; CSF, cerebrospinal fluid.

In order to control for the effects of RSP in relation to CSF log KYNA/KYN ratio and affiliative behavior, we entered the following variables into the GLM: VFD grouping, VFD^∗^RSP interactive term, CSF log KYNA/KYN taken from the second CSF tap (see flow chart in Figure 1), and the VFD^∗^CSF log KYNA/KYN interactive term. The five scores of social affiliative behavior observed during the RSP exposure period served as the repeated measure. CSF log KYNA/KYN ratio positively predicted social affiliative behavior, measured over five separate 2-week blocks (*F*_1_,_28_ = 13.05, *p* = 0.001). However, only in the VFD subjects did CSF log KYNA/KYN ratio positively predict social affiliative behavior, whereas in non-VFD, this relationship was not evident (VFD^∗^log KYNA/KYN interactive effect; *F*_1_,_28_ = 10.21; *p* = 0.0034). For example, in Figure 5, of the five 2-week blocks, the second block shows a positive relationship in VFD between log KYNA/KYN and social affiliation (*r* = 0.82, *N* = 12, *p* = 0.007) in comparison to the corresponding correlation in the non-VFD subjects (*r* = −0.14, *N* = 22, *p* = 0.61). = A repeated-measures^∗^VFD^∗^log KYNA/KYN three-way interactive effect (*F*_4_,_112_ = 5.79; *p* = 0.0003) was observed (see Appendix Table 1). Univariate analyses confirmed that three of the five separate 2-week blocks each revealed VFD^∗^log KYNA/KYN two-way interactive effects while controlling for RSP exposure^∗^VFD interactive effects, indicating that the relationship showed some variability between blocks. The GLM for the second block renders a partial *η*^2^ effect size of 0.50, at least 3.5-fold larger than a large effect size. In VFD, incremental increases in the CSF log KYNA/KYN ratio were associated with a commensurate increase in social affiliative behavior (see Figure 5). The mean of all five 2-week social affiliation tallies obtained during the repeated separation paradigm exposure period and CSF log KYNA/KYN correlate in VFD (*r* = 0.66, *N* = 12, *p* = 0.019).

It remained unclear whether the post-RSP CSF log KYNA/KYN correlation to social affiliative behaviors was indeed dependent on exposure to the repeated separation paradigm as only four of the 12 VFD subjects were in fact exposed to the RSP. We therefore conducted a GLM in which we used as the repeated measure the mean of the baseline three 2-week blocks and then the mean of the five RSP social affiliative behavior 2-week block tallies, categorical variable was whether there was exposure to the RSP paradigm, and CSF log KYNA/KYN derived from the second CSF draw served as the continuous variable. In addition, we used a factorial design that generated a three-way interactive term of repeated measures^∗^CSF log KYNA/KYN^∗^repeated separation paradigm. This term was in fact significant (F_1_,_8_ = 8.70, *p* = 0.018). *Post hoc* Pearson’s correlations revealed that there was indeed a significant positive relationship between CSF log KYNA/KYN and mean social affiliation scores for the RSP-exposed subjects (*r* = 0.98, *N* = 4, *p* = 0.017) versus RSP non-exposed subjects (*r* = 0.14, *N* = 8, *p* = 0.74), supporting the view that the effects were due to the RSP-exposed subjects. There was no significant relationship between the mean baseline scores of social affiliation and CSF log KYNA/KYN derived from the second draw (*r* = 0.36, *N* = 4, *p* = 0.63), indicating that this relationship in RSP-exposed subjects was not due to a pre-existing relationship evident prior to RSP exposure.

The social affiliative behavioral response to the repeated separation paradigm is covered in Appendix Figure 2.

## Discussion

The findings in the current study corroborate our primary hypothesis that early life stress imparts persistent and pervasive changes to the neuro-immune landscape via alterations of KP metabolites. The findings include a reduction of social affiliative behaviors in VFD-reared versus non-VFD control subjects, confirming behavioral alterations in VFD versus non-VFD subjects during baseline home-cage observations (Rosenblum et al., 2001). Reduced baseline CSF log KYNA and lower CSF log KYNA/KYN ratio in VFD-reared subjects in comparison to non-VFD controls is suggestive that early life adversity associates with a relative reduction of neuroprotection alongside increases in KP metabolites within the neurotoxic arm. The aforementioned configuration of the KP metabolites was uniquely evident in VFD-reared subjects. That log KYNA is lower in VFD even when KYN is not indicates reduced activity of key enzymes such as kynurenine aminotransferases (KATs)—which convert KYN to KYNA (Okuno et al., 1991; Parrott et al., 2016b)—specifically in VFD-reared subjects. When controlling for log KYNA/KYN, there were elevations of CSF log anthranilic acid (ANTH) in VFD-reared subjects in comparison to non-VFD controls. The latter result implies an increased activity of KMO in relationship to KAT (Parrott and O’Connor, 2015; Parrott et al., 2016b), which converts kynurenine to 3-hydroxykynurenine and is then converted to 3-hydroxyanthranilic acid by kynureninase. The latter is converted to quinolinic acid by 3-hydroxyanthranilic acid dioxygenase (Krause et al., 2011). An unexpected positive correlation between CSF log KYNA/KYN and CSF log ANTH (Pearson’s *r* = 0.82) is present—uniquely evident in VFD-reared subjects—and is suggestive of a synchronized activity between the neurotoxic and neuroprotective arms of the KP following ELS. The effect size of the VFD-specific synchronization exceeds a large effect size by a factor of three (Cohen, 1973). One plausible explanation that may account for the inordinately high positive correlation between neuroprotective and neurotoxic arms observed in the VFD-reared subjects is a possible coordinated “cross-talk” between the KAT and KMO enzymatic activity.

Following 12 weeks of exposure as adults to the RSP, there are, when compared to the non-VFD no-RSP control group, reductions of CSF log KYNA/KYN obtained from the second blood draw, in RSP-exposed non-VFD. By contrast, for VFD-reared subjects, relative to the no-RSP control condition, CSF log KYNA/KYN is elevated in the RSP condition. The latter two RSP effects erase any non-VFD/VFD effects for CSF log KYNA/KYN post-RSP. Controlling for VFD^∗^RSP effects, behavioral observations of social affiliation scored during the RSP paradigm period show a pattern of correlating positively with CSF log KYNA/KYN obtained following RSP, an effect specifically evident in VFD-reared subjects, but not in non-VFD subjects. Three of the five 2-week blocks of behavioral observations exhibited significant VFD-rearing^∗^log KYNA/KYN interactive effects, indicating, for instance, a Pearson’s *r* = 0.82, specifically in VFD, between social affiliation scores obtained from the second of the five 2-week blocks and CSF log KYNA/KYN. The effect size of the VFD rearing^∗^log KYNA/KYN interactive variable in the prediction of total social affiliation scores is three-fold greater than what is considered a large effect size (Cohen, 1973). Further analyses in VFD reveal that CSF log KYNA/KYN, an index of neuroprotection dependent on the activity of the enzyme KAT, appears intimately related to the social affiliation scores specifically in the four subjects exposed to RSP. The eight no-RSP-exposed VFD subjects neither exhibited a significant correlation nor do the mean social affiliation scores observed during the baseline period prior in the four RSP-exposed VFD subjects.

ELS has been viewed as an independent and preventable risk factor for the elaboration of central hyper-immunity that persists into adulthood (Danese and Baldwin, 2017). This is the first report, to our knowledge, examining central kynurenine pathway (KP) metabolites in non-human primates following the VFD form of early life stress, although the absence of markers of inflammation or central activation of specific cytokines allows for only the suggestion of a persistent hyperimmune state.

Interestingly the effects on KP metabolism in VFD-reared non-human primates did not appear to impact serotonin biosynthesis, which would be expected to occur following extensive and rapid tryptophan diversion to the KP pathway via increased IDO enzymatic activity (Sublette and Postolache, 2012). In this study, both CSF 5-HIAA and CSF tryptophan at baseline did not appear to differ between rearing groups. Previous reports in three VFD-reared cohorts, in fact, have documented high CSF 5-HIAA in VFD-reared subjects, diminishing the likelihood of chronic central tryptophan depletion by high IDO activity (Coplan et al., 2014). The activity of IDO does not appear overtly hyperactivated in VFD as CSF kynurenine concentrations did not differ between the groups. Therefore, the view that early life stress persistently depletes serotonin availability through diversion of tryptophan to the kynurenine pathways and away from serotonin biosynthesis is not supported (Capuron et al., 2002; Capuron et al., 2003). Activation of the KP without an impact on serotonin metabolism is consistent with several related findings in major depressive disorders (Savitz et al., 2015). Future studies may be able to substantiate whether these alterations are a byproduct of low-grade inflammation that may confer vulnerability to depressive disorders and other stress-related disorders (Savitz et al., 2015).

Supporting the view that, following early life stress, CSF log KYNA/KYN ratio is highly relevant to the affective state of macaque subjects, we were able to demonstrate a significant coupling between a compromised neuroprotective profile and social affiliation, an effect specifically observed following the repeated separation paradigm. A separate study aimed at leveraging KMO inhibition is needed to more directly support the social effects of KYNA increases under conditions of chronic stress (Cohen, 1973). That the effect size of the relationship between KYNA/KYN and social affiliation, at its most dramatic, is three-fold greater than a “large effect size” is remarkable as further analyses reveal that this effect is attributable to four RSP-exposed VFD subjects only.

KYNA is produced in astrocytes by KAT and is generally thought to play a neuroprotective role as an allosteric modulator of the glycine site at the NMDA receptor (Guillemin, 2012; Dantzer and Walker, 2014; Schwarcz, 2016). Inhibition of glutamatergic neurotransmission through binding of KYNA to the glycine site of the NMDA receptor was found to decrease striatal dopamine release, with potential consequences for motivational states implicit to social affiliation (Wu et al., 2007). KMO knockout mice maintain the ability to produce KYNA but not QUIN. The KMO knockout prevents accumulation of lipopolysaccharide-induced KP neurotoxic metabolites in the dorsal hippocampus and emergence of depression-like behaviors (Parrott et al., 2016b). The large effect size, seen in the current study, for the two-way interactive effect of VFD^∗^log KYNA/KYN in the prediction of affiliative behavior supports the notion that KP metabolites are sub-serving divergent central functions in ELS versus non-ELS subjects.

The repeated separation paradigm obscures VFD/non-VFD group differences evident in the no-RSP control condition. This effect is true for CSF log KYNA/KYN (see Appendix Figure 1) and social affiliation scores (see Appendix Figure 2), supporting the view that the RSP had significant yet opposing effects on VFD versus non-VFD subjects. Of note, neither the CSF log KYNA/KYN measured after the repeated separation paradigm predict baseline social affiliative behavior nor does baseline KYNA/KYN predict social affiliation during the repeated separation paradigm (data available on request). Thus, the repeated separation paradigm specifically precipitates the emergence of the relationship between KYNA/KYN and social affiliation.

The findings of the current study are consistent with several core domains as defined in the Research Domain Criteria (RDoC) matrix. The current study leverages a paradigm of ELS (in the form of maternal VFD) (Kaufman et al., 2015) and an adult experimental stress paradigm that utilizes aspects of the domain of social cognition (affiliation and attachment construct) (Casey et al., 2014). Finally, as a primary behavioral outcome, social affiliation and its counterpart, social withdrawal, have also been conceptualized within the RDoC framework (Cuthbert, 2019). Social competence has been viewed to depend on functional integrity of three of the RDoC domains: negative valence systems (for example, fear and anxiety in initiating or causing social contact), positive valence systems (for example, reward in response to prosocial behavior), and cognitive systems (Karhson et al., 2016). These convergent domains within the RDoC constructs may enhance replicability and translational value.

Limitations of the study include the examination of only female subjects. It is of note that human plasma KYNA levels are reduced in females and particularly women on oral contraceptives (Meier et al., 2018). It is unclear if these human sex differences translate to macaques. Nevertheless, there is a risk of generating type II errors through inclusion of both sexes when the total number of study subjects is small. Even substantial behavioral and biological differences may not be detected when including sex as a factor because of the small number of subjects within each cell. Nevertheless, adequately powered studies including both sexes are warranted. Because of technical difficulties, we were unable to determine CSF concentrations of QUIN. We were, however, able to measure ANTH, a metabolite of the neurotoxic branch of the KP and a direct, obligate precursor molecule to QUIN. ANTH is converted to QUIN by 3-hydroxyanthranilic acid dioxygenase, an enzyme that does not appear to be influenced by neuroinflammation (Krause et al., 2011). In addition, missing values for various KP metabolites may have led to uneven numbers in the analyses but have nevertheless sufficed to evince highly significant results. The translational significance of reduced social affiliative behavior, at baseline, as an indicator of affective distress in the bonnet macaque may have been inadequately clarified. The repeated separation paradigm, in fact, reduces social affiliation in non-VFD-reared subjects of sufficient magnitude to be rendered indistinguishable from VFD subjects. The reductions of social affiliation in non-VFD exposed to RSP support the view that even contemporaneous adult stress affects patterns of social affiliation.

In summary, ELS is associated with persistent reductions in KP metabolites integral to the neuroprotective arm, such as KYNA, and increases of KP metabolites within the neuroprotective arm, such as ANTH. The collective findings suggest that developmental alterations in neuro-immune activity may extend to the KP in adulthood and may be behaviorally evident in changes of social affiliative behavior. Of note, the underactive neuroprotective arm and overactive neurotoxic arm evident in the current study are paradoxically *positively* correlated specifically in VFD subjects. That these two opposing systems are positively correlated specifically in VFD implies persistent and coordinated cross-talk between the KAT and KMO enzyme systems. Only in VFD does exposure to the repeated separation paradigm reveal a novel relationship between social affiliation and kynurenine metabolites serving neuroprotection. Future studies are needed to establish the presence of perturbed inflammatory status in conjunction with KP metabolite alterations —a model which may serve as an invaluable opportunity for evaluation and development of novel therapeutic options.

## Data Availability Statement

The raw data supporting the conclusions of this article will be made available by the authors, without undue reservation.

## Ethics Statement

The animal study was reviewed and approved by SUNY Downstate, Institutional Animal Care and Use Committee.

## Author Contributions

SAS and JDC drafted manuscript, interpreted data, and critically revised manuscript through submission and acceptance. JDC completed analysis of data. RG, AR, JT, SF, and TP assisted with acquisition of data and made significant contributions to finalizing of manuscript.

## Conflict of Interest

RG was employed by company Firstox Laboratories. The remaining authors declare that the research was conducted in the absence of any commercial or financial relationships that could be construed as a potential conflict of interest.

## Footnotes

1 https://www.aaalac.org/resources/theguide.cfm
