# Supplementary material for: Early Life Stress and the Fate of Kynurenine Pathway Metabolites
Source: Front Hum Neurosci. 2021 Apr 29;15:636144. doi: 10.3389/fnhum.2021.636144 (PMC8117097; doi:10.3389/fnhum.2021.636144)
Supplement: Supplementary file 1 [file Table_1.DOCX]

Appendix:

Appendix Table 1.

*Generalized Linear Model using Factioral ANOVA with Dependent Variable as Mean Social Affiliative Behaviors from Five Two-week Blocks*

| Effect | \| Degr. of Freedom \| \| --- \| | \| F \| \| --- \| | \| P \|  \| \| --- \| --- \| |
| --- | --- | --- | --- | --- | --- | --- | --- |
| \| VFD* RSP \| \| --- \| | **1** | **13.05** | **0.001** |
| \| VFD \| \| --- \| | **1** | **8.57** | **0.0067** |
| \| Log KYNA/KYN \| \| --- \| | **1** | **13.50** | **0.001** |
| \| VFD*Log KYNA/KYN \| \| --- \| | **1** | **10.21** | **0.0034** |
| \| Error \| \| --- \| | 28 |  |  |
| \| R1 \| \| --- \| | 4 | 0.79 | 0.5340 |
| \| R1*VFD*RSP \| \| --- \| | 4 | 1.68 | 0.16 |
| \| R1*VFD \| \| --- \| | **4** | **4.40** | **0.0024** |
| \| R1*Log KYNA/KYN \| \| --- \| | **4** | **4.18** | **0.0034** |
| \| R1*VFD*Log KYNA/KYN \| \| --- \| | **4** | **5.79** | **0.0003** |
| \| Error \| \| --- \| | 112 |  |  |

Legend: Significant tests bolded, VFD = variable foraging demand form of early life stress, RSP = repeated separation paradigm, Log KYNA/KYN = CSF Log Kynurenic acid/kynurenine ratio obtained from the second CSF tap post-repeated seperation paradigm phase (RSP). R1 = Repeated Measures of five consecutive two-week behavioral observation blocks each representing one mean score of up to three observation periods per week. Of the 27 non-VFD subjects available at baseline, data herein is available on 17 of 19 subjects who received the RSP and four of eight who did not receive the RSP. Of twelve VFD, eight of eight subjects assigned to no-RSP and four of four subjects assigned to RSP were available for the current analysis.

Appendix Table 2

Least Square Differences for Appendix Figure 2 of Scatterplot and Error Bars of All Six Subgroups Studied using CSF Log Kynurenic Acid/Kynurenine Ratio

|  | \| {1} 3.07 \| \| --- \| | \| {2} 2.00 \| \| --- \| | \| {3} 1.39 \| \| --- \| | \| {4} 2.80 \| \| --- \| | \| {5} 4.08 \| \| --- \| | \| {6} 3.01 \| \| --- \| |
| --- | --- | --- | --- | --- | --- | --- | --- | --- | --- | --- | --- | --- |
| \| 1 \| \| --- \| |  |  |  |  |  |  |
| \| 2 \| \| --- \| | **0.01** |  |  |  |  |  |
| \| 3 \| \| --- \| | **0.0003** | 0.19 |  |  |  |  |
| \| 4 \| \| --- \| | 0.62 | 0.17 | **0.02** |  |  |  |
| \| 5 \| \| --- \| | **0.05** | **0.00032** | **0.00001** | 0.06 |  |  |
| \| 6 \| \| --- \| | 0.87 | **0.01** | **0.0003** | 0.69 | 0.037 |  |

Legend: The coding in the far left column is as follows: 1) Non-VFD pre-RSP (N=14), 2) VFD pre-RSP (N=10), 3) VFD RSP (N=4), 4) VFD No-RSP (N=8), 5) non-VFD no-RSP (N=5), 6) non-VFD RSP (N=17).

Appendix Table 3.

Missing Values -Baseline Comparisons

| Variable | Non-VFD (N= 27) | VFD (N=12) |
| --- | --- | --- |
| Baseline Affiliation * | 25 | 12 |
| Log KYNA | 15 | 10 |
| Log KYNA/KYN | 14 | 10 |
| Log ANTH/KYN | 14 | 9 |

*Three separate two-week observations

Missing Values-Response to Repeated Separation Paradigm (RSP)

| Log KYNA/KYN | VFD-RSP (N=4) | VFD – No RSP (N= 8) | Non-VFD RSP (N=19) | Non-VFD No RSP (N=8) |
| --- | --- | --- | --- | --- |
| Baseline | 4 | 6 | 14 | 0 |
| RSP | 4 | 8 | 17 | 5 |

*Appendix Figure 1. The Effects of the Repeated Separation Paradigm on CSF Log KYNA/KYN as a Function of VFD exposure during Early Life*


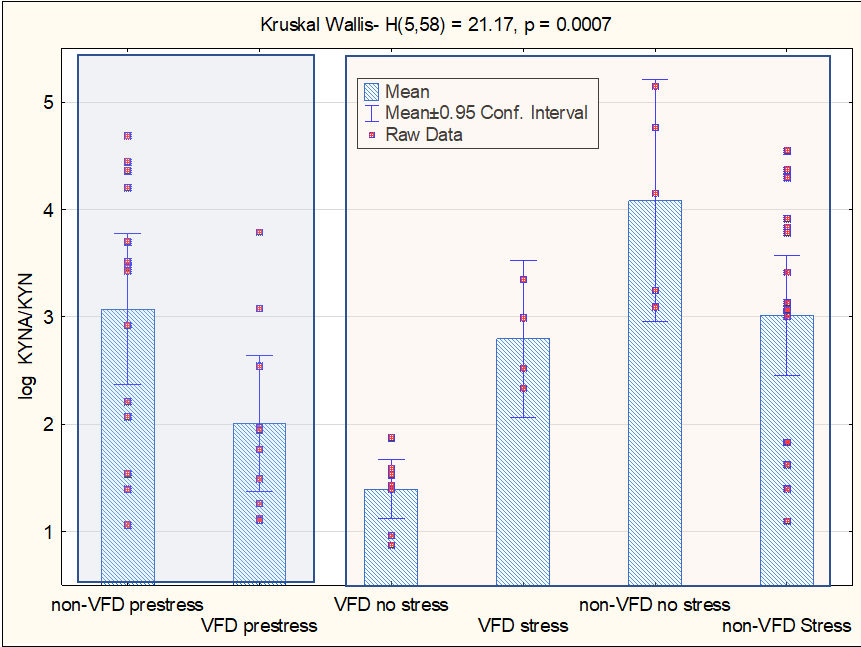


“Stress” in the legend refers to exposure the “repeated separation paradigm”. Values from the two left hand columns shaded in blue represent values from the first CSF tap versus the four columns on the left-hand side shaded in red represent the values from the second tap following the RSP exposure/No-exposure phase. A generalized linear/non-linear model was used assigning each of the above six groups an ascending code as the categorical variable and using log KYNA/KYN as the dependent variable for 54 values. An overall effect of grouping was noted [Wald statistic _5_ = 27.43, p = 0.000047]. As can be seen from post-hoc least square differences in Appendix Table 2, there is a significantly higher log KYNA/KYN value for the non-VFD no-RSP (N = 5) and non-VFD pre-RSP (N = 14) compared to the remainder of subjects. However, the range of values of non-VFD no RSP are not dramatically divergent from the overall values. VFD RSP exposure (N = 4) is elevated in comparison to VFD no-RSP (N = 8). The non-VFD RSP (N=17) is in fact significantly lower than non-VFD pre-RSP (N = 14) suggesting an effect of RSP on log KYNA/KYN in the downward direction.

Appendix Figure 2:

*Effects of the Repeated Separation Paradigm on Social Affiliation Behavior as a Function of Early Life Stress*

Legend: Social Affiliative Scores are determined from the mean of five consecutive two-week behavioral observation blocks during a 12 week repeated separation paradigm (RSP). An overall VFD effect was noted in the GLM model, which controlled for CSF log KYNA/KYN, for mean behavioral affiliation in response to RSP [F_1,28_= 8.56, p = 0.007]. However, the rearing effects are most prominent when there is no RSP exposure in comparison to RSP exposure [Rearing group* RSP exposure: F_1,28_= 13.05, p = 0.001]. In fact, behavioral affiliation in non-VFD is numerically lower (N = 17) than VFD values both in response to the RSP (N = 4) with the caveat that the means are adjusted to covariates. VFD No-RSP (N = 8), Non-VFD No RSP (N = 4).

Appendix: Chemical Extraction

To assure sensitivity and eliminate chromatographic interferences, a simple chemical extraction of 100ul of CSF from each of the collection timepoints was put through a simple protein crash chemical extraction. 100ul of thawed CSF was placed into a borosilicate test tube along with 50ul of 20ng/ml internal standard, and 2mls of LC-MS/MS grade acetonitrile. The sample was mixed and then centrifuged for ten minutes at 12,000 x g in an Eppendorf 5804 model centrifuge that was chilled at 10C. The solution was transferred avoiding the protein layer into a new tube and evaporated under nitrogen until completely dry. Each sample was then reconstituted with 20ul of LC-MS/MS methanol and 180ul of 18 ohm purity water. Samples were placed in vials and 20ul was injected.

LC-MS/MS

LC-MS/MS is commonly viewed as the “gold standard’ of analytical tools because of sensitivity, selectivity, specificity and reproducibility of results (CLSI, 2007). Sciex 6500+ tandem mass spectrometer was used with an electrospray ionization source coupled to an Agilent Infinity II 1290 UHPLC for separation and detection of analytes.

An internal standard is a molecule that cannot be made biologically and that is added to a sample at a known concentration to assure accurate and precise quantitation. Typically, when LC-MS/MS is used, a deuterated internal standard is utilized. Deuterated internal standards are molecules that have hydrogens replaced with heavy hydrogens, deuterium, that have a neutron and a proton, therefore affecting the overall molecular mass without affecting the affinity and ionization of that molecule. The use of LC-MS/MS presented one major analytical issue: deuterated internal standards of the various kynurenine pathway metabolites were unobtainable. Surrogate internal standards were selected to be amphetamine and morphine obtained from Cerialliant™. These internal standards were chosen not only for availability but also due to similarities in molecular weight and column affinity when compared to target analytes. The concentration of an unknown sample was determined using the Multiquant™ 3.0.2 software, which calculates the following quantitation equation automatically:

*Calibrator Analyte Area/ Internal Standard Area = Standard Factor Ratio*

*Linear curve is plotted with X= calibrator concentration, Y= Standard Factor Ratio*

*Unknown Analyte Area/ Internal Standard Area = Unknown Factor Ratio*

*Unknown Factor Ratio is then plotted on curve to determine quantitation.*

External standard was used for anthranilic acid in place of an internal standard. An external standard is an outside known solution that is used to determine peak area. From this known peak area, a simple calculation can be done to determine analyte concentration of an unknown. As an example;

*Unknown Concentration = (Peak Area of Unknown Sample)/(peak area of 1ng/ml standard)*

(1)Linear ranges as follows; kynurenine = 1-100 ng/ml, kynurenic acid = 10-1000 ng/ml, 5hiaa = 10-500 ng/ml, tryptophan = 100 – 1000 ng/ml, and anthralinic acid = 0.1 – 100 ng/ml.

DATA PROCESSING

Chromatographic data was processed with Multiquant™ 3.0.2 software with a gaussian smoothing of three for all ions. To be considered a true positive, a three-factor set of criteria was used which included presence of two transitions, retention time based from the calibrator, and ion ratio within 20%. Anthranilic acid is the only exception because one transition was used instead of two, due to neutral fragment loss so therefor ion ratio was not utilized.
